# Anti-Aβ3–10 monoclonal antibody 7B8 improves cognitive function and protects the blood-brain barrier in APP/PS1 mice by regulating the HMGB-1/RAGE/NF-κB pathway

**DOI:** 10.3389/fimmu.2026.1781351

**Published:** 2026-04-30

**Authors:** Jiayu You, Qianshuo Liu, Xingqiang Li

**Affiliations:** 1 Department of Neurology and Neuroscience, Shenyang First People’s Hospital, Shenyang Brain Institute, Shenyang, Liaoning, China; 2 Department of Neurology, the Fourth Affiliated Hospital of China Medical University, Shenyang, Liaoning, China

**Keywords:** 7B8 monoclonal antibody, Alzheimer’s disease (AD), amyloid-related imaging abnormalities (ARIA), APP/PS1 double-transgenic mice, Aβ deposition, blood-brain barrier (BBB), cognitive function, glial cell activation

## Abstract

**Background:**

Alzheimer’s disease (AD), the most prevalent dementia, is primarily underpinned by the amyloid cascade hypothesis. Passive Aβ immunotherapy effectively reduces cerebral Aβ deposition but is limited by severe side effects, including cerebral amyloid angiopathy (CAA), microhemorrhage, and amyloid-related imaging abnormalities (ARIA). Here, we investigated the efficacy and safety of a novel anti-A_3–10_ monoclonal antibody (7B8) in APP/PS1 double-transgenic mice, with a focus on its impacts on amyloid clearance, neuroinflammation, and blood-brain barrier (BBB) integrity.

**Methods:**

7B8 was generated by immunizing mice with A_3–10_-KLH. Six-month-old APP/PS1 mice were intraperitoneally injected with 7B8 (10 mg/kg) weekly for 8 doses (7B8 group). Age-matched APP/PS1 mice treated with IgG and C57BL/6J mice served as negative and wild-type (WT) controls, respectively. One week after the final injection, behavioral tests were performed, followed by euthanasia for histological (left brain hemisphere) and biochemical (right brain hemisphere) analyses.

**Results:**

Compared with the IgG group, the 7B8 group exhibited significantly reduced cerebral Aβ deposition and improved cognitive function (both *P* < 0.05), comparable to the WT group. Notably, in these young 6-month-old APP/PS1 mice with early-stage amyloid deposition and minimal CAA pathology, 7B8 treatment did not increase microhemorrhage risk relative to the IgG control group (P > 0.05). Furthermore, 7B8 preserved vascular integrity by reducing perivascular Aβ_40_ deposition and smooth muscle actin damage, while enhancing endothelial cell fluorescence intensity (*P* < 0.05). At the molecular level, 7B8 upregulated vascular LRP-1 and BBB tight junction proteins (ZO-1, CLDN-5, Occludin), and downregulated RAGE expression (*P* < 0.05). It also suppressed microglial and astrocytic activation, reduced levels of IL-6 and cortical TNF-α, and inhibited the HMGB-1/RAGE/NF-κB signaling pathway (*P* < 0.05), without affecting global TNF-α or IL-1β levels.

**Conclusion:**

7B8 effectively alleviates cognitive impairment and clears cerebral and perivascular amyloid deposits in young APP/PS1 mice with early-stage AD pathology and minimal CAA, with no increased risk of microhemorrhage in this experimental setting. It also protects vascular structure and BBB integrity by inhibiting the HMGB-1/RAGE/NF-κB-mediated neuroinflammatory response. Given the limitations of evaluating CAA-related safety in young mice, future studies using mid-aged (12–15-month-old) APP/PS1 mice with prominent CAA will be conducted to fully characterize 7B8’s safety profile. These findings highlight 7B8 as a promising candidate for safe and effective AD immunotherapy, providing new insights into the development of ARIA-minimizing strategies.

## 1 Introduction

Alzheimer’s disease (AD) is a progressive neurodegenerative disorder characterized by core clinical manifestations including memory impairment, cognitive decline, and language dysfunction, and represents the most prevalent form of dementia. Amid the global aging demographic shift, AD has emerged as one of the most costly and life-threatening diseases worldwide (1). Globally, approximately 57 million individuals are living with dementia, of which AD accounts for roughly 32 million cases. Experts predict this figure will surge to 153 million by 2050, exerting an overwhelming health, social, and economic burden on societies and healthcare systems globally (2).

AD’s core neuropathological hallmarks involve two primary pathological processes: extracellular amyloid-β (Aβ) protein accumulation leading to senile plaque (SP) formation, and intracellular hyperphosphorylation of tau protein followed by aggregation into neurofibrillary tangles (NFTs) (3). Beyond these two signature pathologies, histological studies consistently demonstrate neuronal loss and cerebral amyloid angiopathy (CAA) as additional key pathological manifestations (4). Notably, the “amyloid cascade hypothesis” posits that the pathogenesis and progression of AD are primarily driven by a single core mechanism (5).

Guided by the amyloid cascade hypothesis, researchers have vigorously pursued Aβ-targeting therapies—including anti-Aβ immunotherapies—to intercept the pathogenic process of AD at an early stage (3). However, these therapeutic strategies encountered a prolonged period of setbacks, raising widespread doubts about the validity of the amyloid cascade hypothesis (6). Aducanumab was the first anti-Aβ monoclonal antibody to receive FDA accelerated approval in 2021 for early-stage AD, owing to its ability to clear cerebral parenchymal and vascular Aβ aggregates. However, it was subsequently withdrawn from global development and commercialization by Biogen in 2023, with the EMA having already rejected its marketing authorization in 2021 (7). Two pivotal scientific reasons underpinned this withdrawal: first, inconsistent Phase 3 trial data cast doubt on the clinical benefit of Aβ clearance—its high-dose regimen showed mild cognitive improvement in one trial but no significant efficacy in another, failing to confirm a definitive link between Aβ reduction and cognitive preservation. Second, severe amyloid-related imaging abnormalities (ARIA) (including vasogenic edema and microhemorrhage) posed major safety risks, with APOEϵ4 carriers exhibiting drastically elevated susceptibility; neuropathological evidence further confirmed complement activation and microinfarcts in CAA-laden vessels of ARIA-positive regions in treated patients (8).

A pivotal breakthrough subsequently emerged: the U.S. Food and Drug Administration (FDA) has recently approved two anti-Aβ immunotherapies, lecanemab and donanemab, representing a significant milestone in anti-AD research (9). Notably, these agents exhibit remarkable efficacy: when administered at optimal doses, both achieve substantial Aβ plaque clearance—even approaching near-complete elimination—and exert a moderate inhibitory effect on cognitive decline (10). This transition from repeated failures to tangible progress is highly informative, underscoring that Aβ targeting remains a promising therapeutic direction despite the arduous path to advancement. Nevertheless, the occurrence of ARIA in a subset of patients necessitates careful risk mitigation, rigorous monitoring, and prudent patient selection (11). ARIA is presumably multifactorial in the context of passive immunization, with key contributing factors including perivascular amyloid overload, glial cell overactivation, and excessive production of inflammatory cytokines (12). Beyond CAA and microhemorrhage, ARIA induced by passive Aβ immunization is also associated with complement activation (e.g., C3 and C5b-9 deposition) (13), as activated astrocytes and microglia—key components of the neuroinflammatory microenvironment—are the primary sources of complement proteins. A retrospective clinical study further identified microinfarcts with hemosiderin and complement activation in ARIA-positive regions on MRI (14), highlighting complement as a critical mediator of ARIA pathology. These factors are thought to interact synergistically rather than act independently, although the precise nature of their crosstalk remains incompletely elucidated in the current literature.

Amyloid deposits can be transported from the brain parenchyma to vessel walls, and with prolonged accumulation, these deposits displace the inherent structural components of the vessel wall. This, in turn, impairs the vessel’s normal contractile and pulsatile function (15). Notably, glial cell activation and inflammatory responses represent a classic double-edged sword in AD pathogenesis. Activated glial cells can facilitate amyloid clearance and phagocytosis in the early stages, which constitutes a protective role. However, sustained and chronic glial activation ultimately induces neuronal and synaptic damage, accelerating the progression of AD (16). Furthermore, perivascular inflammation may compromise the integrity of the blood-brain barrier (BBB), resulting in blood extravasation.

In the context of passive immunotherapy, while these agents effectively promote amyloid clearance, they also inevitably trigger glial cell activation. The key challenge arises when such activation becomes excessive: overstimulated glial cells secrete elevated levels of inflammatory cytokines. Accumulating evidence indicates that these cytokines can damage the vascular wall structure, microvascular endothelial cells, and the transport function of Aβ within the BBB and neurovascular unit (NVU) (17). Collectively, these pathological changes contribute to fluid or blood extravasation, which is a critical pathological basis for the development of ARIA. Although the underlying mechanism constitutes a complex cascade, the current evidence supports this sequential pathological pathway, which remains to be fully elucidated.

Thus, there is an urgent need for novel monoclonal antibodies capable of effectively clearing Aβ deposits from the brain parenchyma, reducing vascular wall-associated amyloid deposition, alleviating cognitive impairment, and—crucially—without increasing the risk of microhemorrhage. Notably, given the persistent ARIA-related concerns surrounding current passive immunotherapies, achieving this balance between therapeutic efficacy and safety has become increasingly imperative. To clarify the association between ARIA and passive immunotherapy in AD, our team evaluated a novel anti-Aβ3–10 monoclonal antibody using APP/PS1 double-transgenic mice, with two core objectives: first, to assess its efficacy in clearing both parenchymal and perivascular Aβ deposits; second, to investigate its effects on BBB integrity and inflammatory responses. This targeted investigation aimed to address critical gaps in current therapeutic strategies for AD.

Building upon our prior research, we have confirmed that the Aβ3–10 vaccine induces therapeutic levels of antibodies and effectively eliminates SPs from the brain parenchyma (18–22). In the present study, monoclonal antibodies were generated from mice immunized with the Aβ3-10-KLH conjugate vaccine; as demonstrated in our earlier work, these antibodies exhibit specific binding affinity for Aβ oligomers and fibrils (23). APPswe/PSEN1dE9 (APP/PS1) transgenic mice were employed as the AD model, owing to their well-characterized pathological features and progressive cerebral Aβ deposition (24). This model typically exhibits parenchymal Aβ plaque deposition in the brain at approximately 4 months of age; by 6 months, mild perivascular Aβ accumulation can be detected, yet CAA pathology remains extremely minimal at this age, and these mice do not develop spontaneous microhemorrhages during this developmental stage (25, 26). Six-month-old APP/PS1 mice were treated with the anti-Aβ3–10 monoclonal antibody at a dosage of 10 mg/kg, with a total of 8 administrations. The primary objective of this study was to evaluate the antibody’s effects on both parenchymal and perivascular Aβ deposition.

## 2 Results

### 2.1 Antibody 7B8 reduced Aβ deposition in brain parenchyma

**Figure 1:**
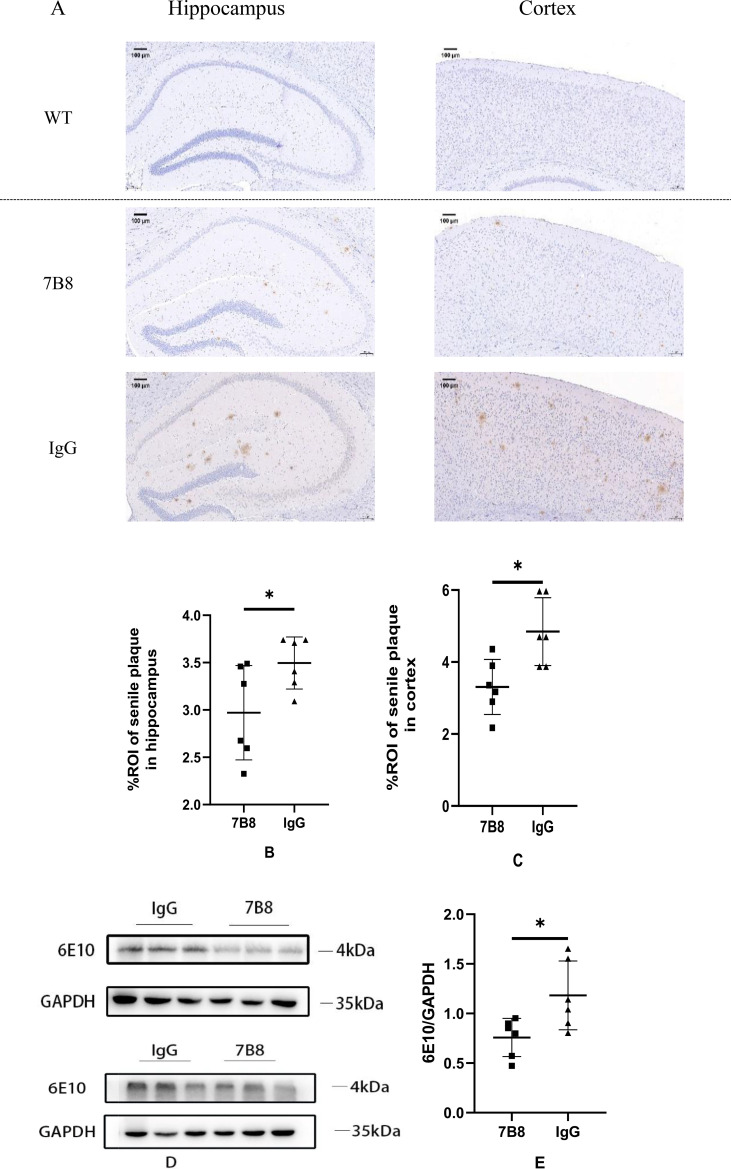
Immunohistochemical staining and WB of Aβ in mouse brain tissue. **(A–C)** Immunohistochemical staining of Aβ deposition in the hippocampal and cortical regions of APP/PS1 mice (IgG vs 7B8 groups). Quantitative analysis of Aβ plaque area was performed, with statistical comparisons only between the IgG and 7B8 groups (n=6, *P<0.05). (scale: 100μm). **(D, E)** Western blot analysis and quantitative results of human Aβ protein levels in the brain tissues of APP/PS1 mice (IgG vs 7B8 groups). GAPDH was used as the internal reference, with statistical comparisons only between the IgG and 7B8 groups (n=6, *P<0.05).

Immunohistochemical (IHC) staining revealed that Aβ deposition in the hippocampal and cortical regions was significantly reduced in the 7B8 group relative to the IgG control group, with statistically significant differences observed (n=6, *P<0.05) (**Figures 1A–C**). The wild-type (WT) group was included solely as a negative physiological control, and only an extremely weak immunopositive signal was detected in this group—this signal does not represent authentic senile plaques, but rather non-specific cross-reactivity of the 4G8/6E10 antibodies with endogenous murine amyloid precursor protein (APP), rather than extracellular human Aβ deposition. In **Figures 1A–C**, the WT group is visually separated from the experimental groups (IgG and 7B8) with a dotted line for clear distinction, and no statistical comparisons were performed between the WT group and the two experimental groups. Consistent with the IHC findings, Western blot (WB) analysis further confirmed that the 7B8 antibody significantly decreased human Aβ levels in the brain tissues of APP/PS1 mice compared with the IgG control group (n=6, *P<0.05), (**Figures 1D, E**). WB analysis also failed to detect human Aβ protein signals in WT mice, further confirming the specificity of the detection method for human Aβ. Collectively, these results demonstrate that the 7B8 antibody effectively inhibits parenchymal Aβ deposition in the brain of APP/PS1 mice, which is consistent with its predicted therapeutic activity.

### 2.2 Antibody 7B8 reduced Aβ deposition around vascular walls

**Figure 2:**
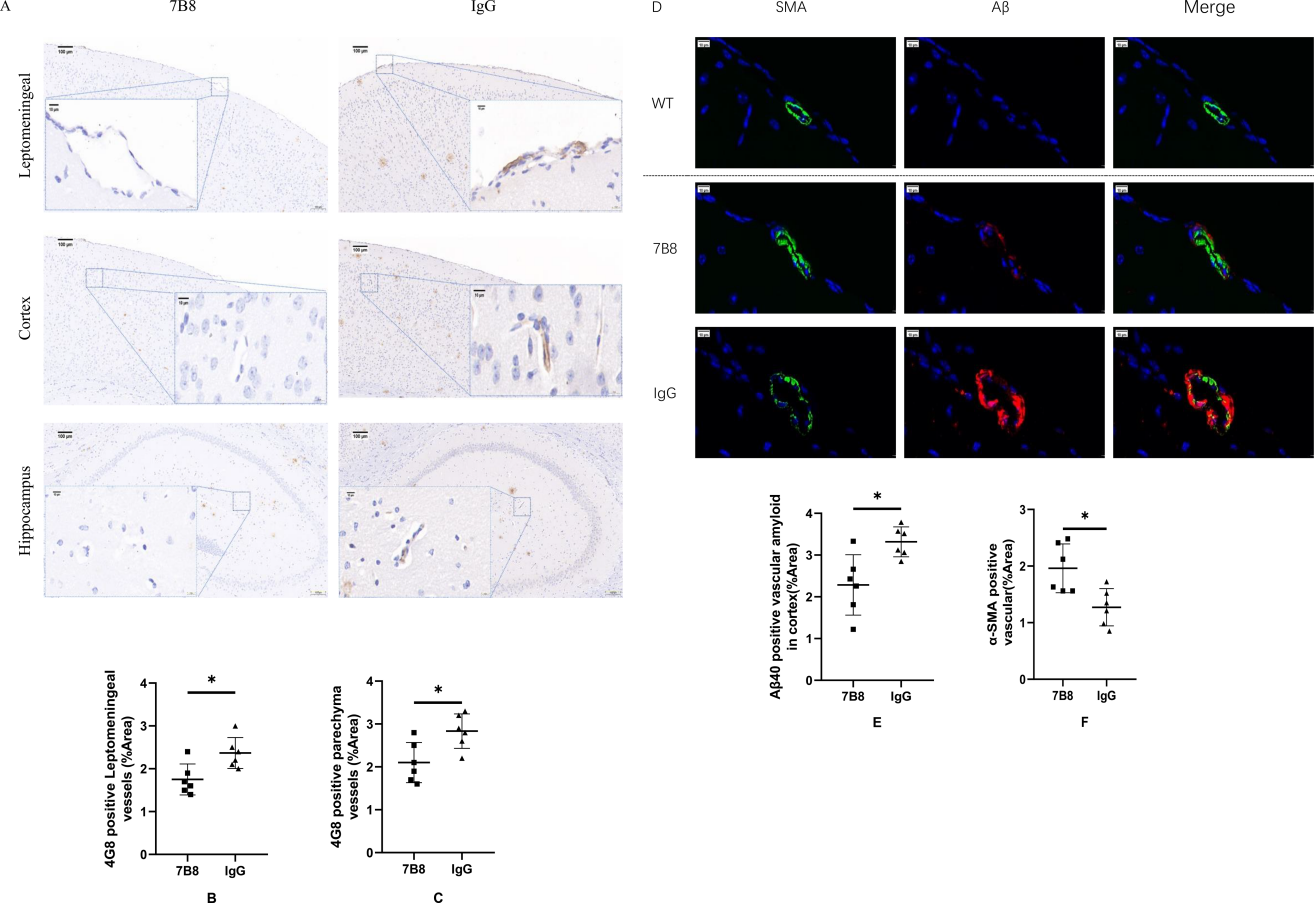
IHC and IF staining of Aβ deposition around the vessel wall. **(A–C)** IHC staining of perivascular Aβ deposition in cerebral blood vessels of APP/PS1 mice (IgG vs 7B8 groups). Quantitative analysis of perivascular Aβ-positive area was performed with statistical comparisons only between the IgG and 7B8 groups (n=6, *P<0.05). (scale: 100μm and 10μm). **(D–F)** IF staining and quantitative analysis of α-SMA expression in vascular smooth muscle cells of APP/PS1 mice (IgG vs 7B8 groups). α-SMA serves as a marker for vascular smooth muscle cell integrity; statistical comparisons were only performed between the IgG and 7B8 groups (n=6, *P<0.05). (scale: 10μm).

Vessel-associated amyloid deposition was quantified by defining perivascular Aβ as Aβ deposits located within 50 μm of the vascular wall. A consistent threshold algorithm was applied to measure the percentage of Aβ-positive area, the number of CAA-affected vessels, and glial cell density. Quantification was performed on 3–4 consecutive coronal brain sections per mouse, with a mean of 25 ± 3 vessels (minimum 22 vessels) analyzed per animal. Values were averaged per mouse for subsequent statistical analysis. This standardized sampling strategy ensured robust statistical power to detect subtle differences in CAA burden, even at this early age (6–8 months) when vascular amyloid deposition was limited. IHC staining showed that perivascular Aβ deposition was significantly lower in the 7B8 group compared to the IgG control group (n=6, *P<0.05) (**Figures 2A–C**). WT mice were included as a negative physiological control in this study, and no detectable vessel wall-associated Aβ deposition was observed in WT mice via IHC staining, which was consistent with the physiological features that WT mice lack human APP/PS1 transgenes and do not produce or accumulate human Aβ. Consistent with this finding, immunofluorescence (IF) staining further corroborated a marked reduction in vessel wall-associated Aβ in the 7B8 group relative to the IgG control group. Furthermore, IF staining revealed an additional key observation: the expression levels of vascular smooth muscle actin (α-SMA)—a marker of vascular smooth muscle cell integrity—were significantly higher in the 7B8 group than in the IgG control group (n=6, *P<0.05) (**Figures 2D–F**); WT mice exhibited constitutively high physiological expression of α-SMA in vascular smooth muscle cells, serving as a reference for normal vascular wall integrity. Collectively, these results indicate that the 7B8 antibody protects vascular wall integrity by reducing perivascular Aβ deposition. This observation is biologically plausible, as diminished amyloid accumulation around blood vessels would preserve the structural integrity of vessel walls and maintain the expression of vascular smooth muscle proteins. These findings support the predicted vascular protective effect of the 7B8 antibody.

### 2.3 Antibody 7B8 alleviated neuronal damage in brain tissue

**Figure 3:**
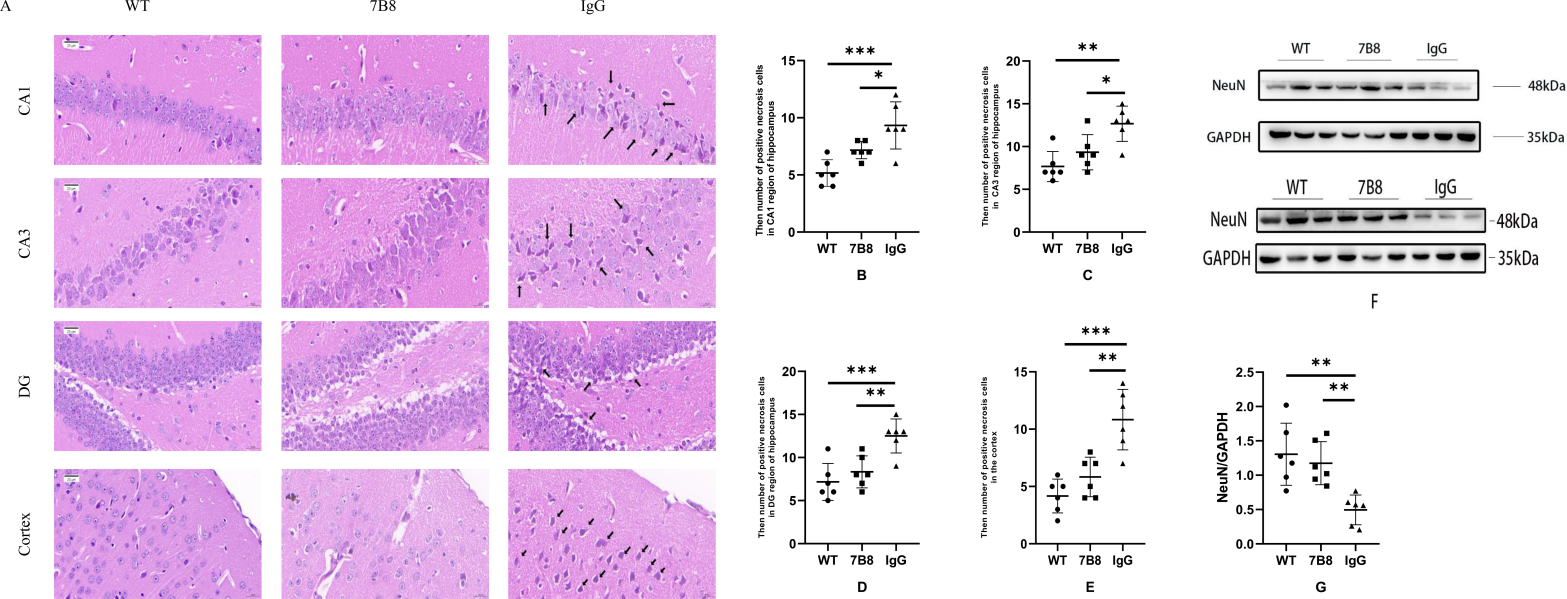
Therapeutic effect of 7B8 on hippocampal and cortical neuronal loss and death in APP/PS1 mice. **(A)** HE staining of the hippocampal CA1, CA3, DG regions and cortex (scale bar: 20 μm). **(B–E)** Compared with the IgG control group, neuronal necrosis in the hippocampal CA1, CA3, DG regions and cortical area was significantly decreased in the 7B8 group, with statistical differences (n=6, *P<0.05, **P<0.01, ***P<0.001). The WT group was included as a negative physiological control, and no significant neuronal necrosis was observed in WT mice (consistent with normal neuronal integrity in non-transgenic mice). **(F)** WB analysis of NeuN protein expression in the WT group, IgG control group, and 7B8 group. **(G)** NeuN protein levels in the IgG control group were significantly lower than those in the 7B8 group (n=6, *P<0.05, **P<0.01, ***P<0.001). The WT group exhibited constitutively high physiological expression of NeuN, serving as a reference for normal neuronal density.

Neuronal necrosis was identified as the primary form of neuronal damage via hematoxylin-eosin (HE) staining, consistent with classical morphological criteria for neuronal necrosis in brain tissue. The severity of neuronal necrosis was significantly alleviated in both the 7B8 and WT groups compared to the IgG control group, with statistically significant differences (n=6, *P<0.05, **P<0.01, ***P<0.001) (**Figures 3A–E**). To distinguish neuronal necrosis from other cell death subtypes (e.g., apoptosis), key morphological features in HE-stained sections were analyzed: neurons in the IgG control group exhibited hallmark necrotic characteristics, including cell body swelling, cytoplasmic membrane disruption, eosinophilic cytoplasmic condensation, and irregular nuclear fragmentation. In contrast, apoptotic features (e.g., cell shrinkage, intact cytoplasmic membranes, or apoptotic bodies) were not observed. These findings indicated that the 7B8 antibody mitigates neuronal necrosis, which is consistent with our previous observation that 7B8 clears Aβ deposits—a well-documented trigger of necrotic neuronal loss in APP/PS1 mice.

Notably, NeuN (neuronal nuclei antigen) is a validated marker for mature, viable neurons. Complementary WB analysis corroborated the morphological observations: NeuN protein expression levels were significantly higher in the WT and 7B8 groups than in the IgG control group (**Figure 3F**), with statistically significant differences (n=6, *P<0.05, **P<0.01, ***P<0.001) (**Figure 3G**). This result demonstrated that the 7B8 antibody preserves the population of mature neurons, which correlates with the reduced neuronal necrosis observed in morphological analyses.

Collectively, these data support the notion that the 7B8 antibody exerted a neuroprotective effect, likely mediated by its ability to reduce Aβ accumulation and subsequent necrotic neuronal damage. While the identification of neuronal necrosis is based on well-characterized morphological criteria in HE-stained sections, we acknowledge that complementary molecular assays (e.g., immunostaining for necrosis-specific markers such as HMGB1, or TUNEL assay for apoptosis) could further validate the cell death subtype in future studies. Currently, the consistency between attenuated necrotic morphology and preserved NeuN expression supports this interpretation.

### 2.4 Antibody 7B8 reduced cognitive impairment

**Figure 4:**
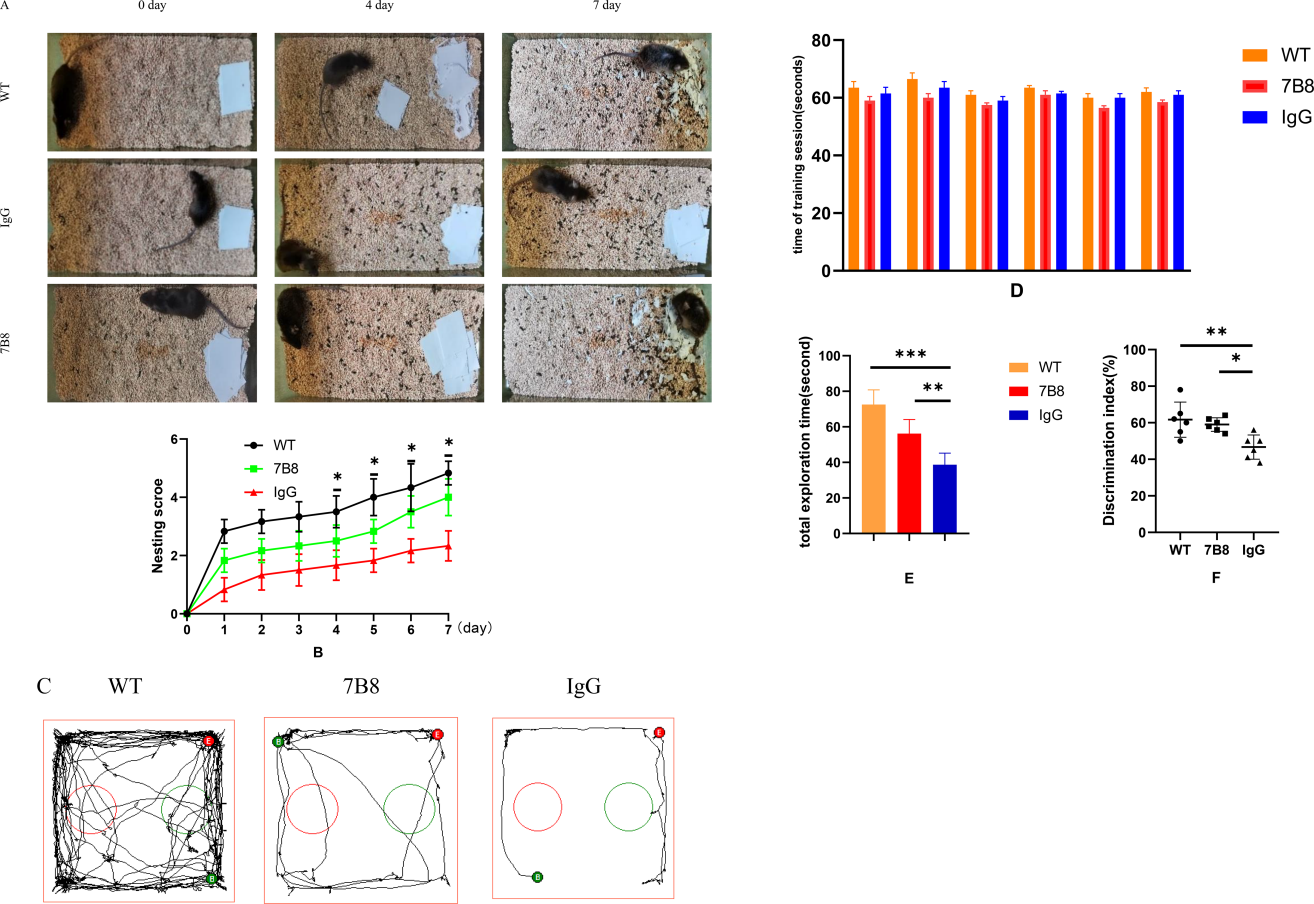
Effect of antibody 7B8 on cognitive function in APP/PS1 mice. **(A, B)** The nestlet shredding test showed that antibody 7B8 increased the fossa score (n=6, *P<0.05). **(C)** Representative movement tracks of WT, IgG and 7B8 mice in the NOR training session. **(D)** Exploration time for left and right identical objects in the training session of the NOR test **(E)** The total exploration time of all groups in the test phase revealed that the WT and 7B8 groups had a significantly longer exploration time than the IgG group (n=6, **P<0.01, ***P<0.001). **(F)** The NOR test showed that the antibody 7B8 improved the resolution index (n=6, *P<0.05, **P<0.01).

To evaluate the effect of the 7B8 antibody on cognitive function in APP/PS1 mice, two well-validated behavioral tests for assessing rodent recognition and memory were employed (27): the nestlet shredding test (**Figure 4A**) and the novel object recognition (NOR) assay (**Figure 4C**). Briefly, the nestlet shredding test primarily assessed nest-building ability (associated with natural behavior and cognitive function), while the NOR assay evaluated short-term memory and learning capacity. Data from the training session were provided to confirm equal initial exploration of the two identical objects and validate the absence of inherent side-bias in experimental mice (**Figure 4D**). Paired t-test analysis revealed no significant differences in exploration time between the left and right objects within any of the three groups: the WT group (t=0.92, df=5, P = 0.39), IgG group (t=0.75, df=5, P = 0.48), and 7B8 group (t=0.88, df=5, P = 0.41; all P > 0.05). These results demonstrated that the mice showed no innate side-bias and explored the two objects equally at the initial training stage, thereby validating the methodological reliability of the training phase in the NOR assay. The total exploration time of all groups during the test phase was above the normal threshold (>20 s), with significant group differences observed (one-way ANOVA, F(2,15)=12.65, P<0.001): the WT group (72.5 ± 8.3 s) had the highest total exploration time, followed by the 7B8 group (56.2 ± 7.9 s) and the IgG group (38.7 ± 6.5 s) (n=6 per group, P<0.05 for WT vs IgG/7B8, P<0.05 for 7B8 vs IgG, **Figure 4E**). Pearson correlation analysis further confirmed no significant correlation between the novel object recognition index and total exploration time in the 7B8 group (r=0.23, P = 0.38), excluding confounding effects of non-cognitive factors (e.g., exploratory activity) on the cognitive test results.

Behavioral test results revealed significant improvements: the 7B8 antibody significantly enhanced both the nest-building score (n=6, P<0.05) (**Figure 4B**) and the discrimination index (DI) in the NOR assay (n=6,*P<0.05, **Figure 4F**). These findings indicated that the 7B8 antibody alleviates cognitive impairment in APP/PS1 mice. Notably, this functional improvement links the Aβ-clearing efficacy of the 7B8 antibody (demonstrated in previous results) to functional cognitive improvements rather than merely structural changes.

### 2.5 Antibody 7B8 did not increase cerebral microhemorrhages

**Figure 5:**
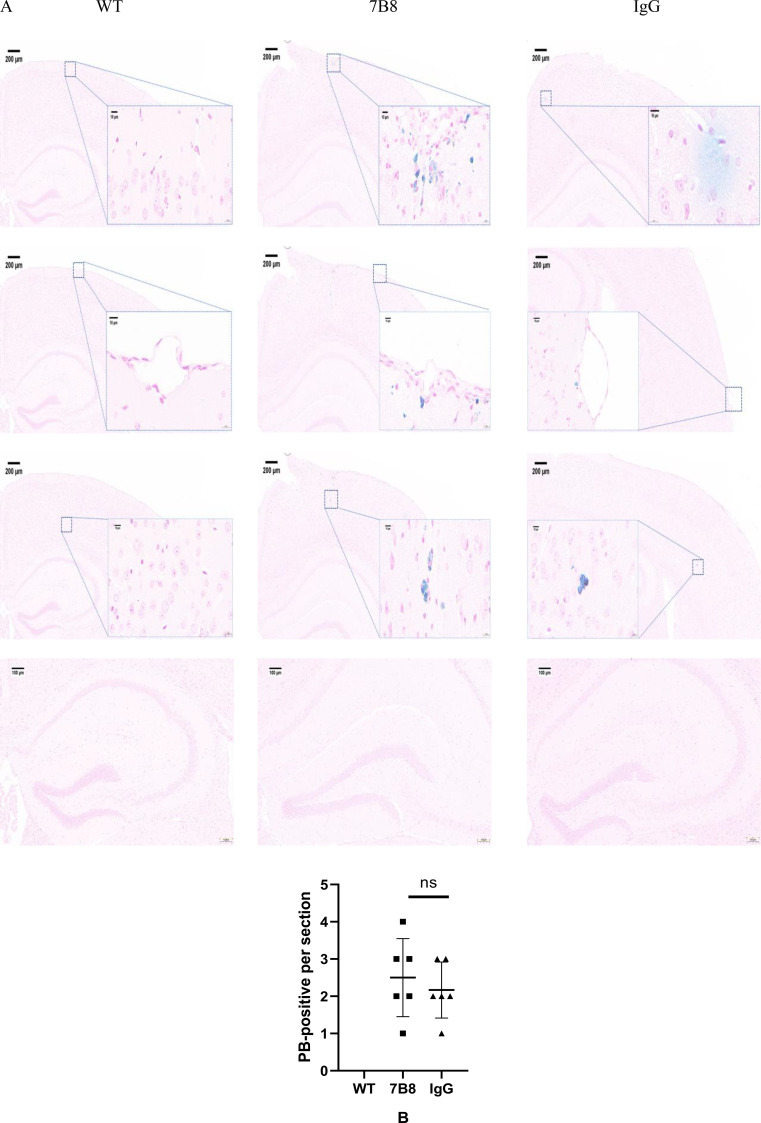
PB staining detected microhemorrhage in mouse brain tissue (scale: cortex 200μm, hippocampus 100μm). **(A)** PB staining results showed that microhemorrhage aggregation was concentrated in the cortex, subcortical and leptomeningeal vessels of brain tissue in group 7B8 and IgG group. **(B)** There was no significant difference in the number of positive PB staining between the 7B8 group and the IgG group (n=6, P>0.05).

Given that APP/PS1 transgenic mice at 6 months of age exhibited only minimal CAA pathology, Prussian blue (PB) staining revealed sparse microhemorrhages in both the 7B8 and IgG control groups, which were predominantly distributed in the subcortical brain tissue and around leptomeningeal vessels; no PB-positive signals were detected in the hippocampus of either group (**Figure 5A**). Further regional analysis showed no obvious differences in PB-positive signals between areas adjacent to pial vessels and other brain regions. The 7B8 group showed a slight increase in PB positivity compared with the IgG control group, but the difference was not statistically significant (n=6, P>0.05) (**Figure 5B**).

We did not perform combined vascular amyloid and microhemorrhage staining (Prussian blue + Thioflavin for example), which is required to directly verify the relationship between CAA and microhemorrhages. Therefore, the low and non-significant difference in microhemorrhages should not be overinterpreted as a favorable vascular safety profile of 7B8, but is most likely due to the minimal baseline CAA burden in 6-month-old mice. This study would benefit from a larger sample size. Future studies using 15-month-old APP/PS1 mice or other models with moderate-to-severe CAA will be necessary to more rigorously and convincingly evaluate the vascular safety of 7B8.

### 2.6 Antibody 7B8 protected endothelial cells

Brain microvascular endothelial cells are central to maintaining the integrity and function of the BBB. To determine whether the 7B8 antibody protects these cells in APP/PS1 mice, immunofluorescence staining of brain tissue sections was performed using CD31—a well-validated marker for microvascular endothelial cells.

**Figure 6:**
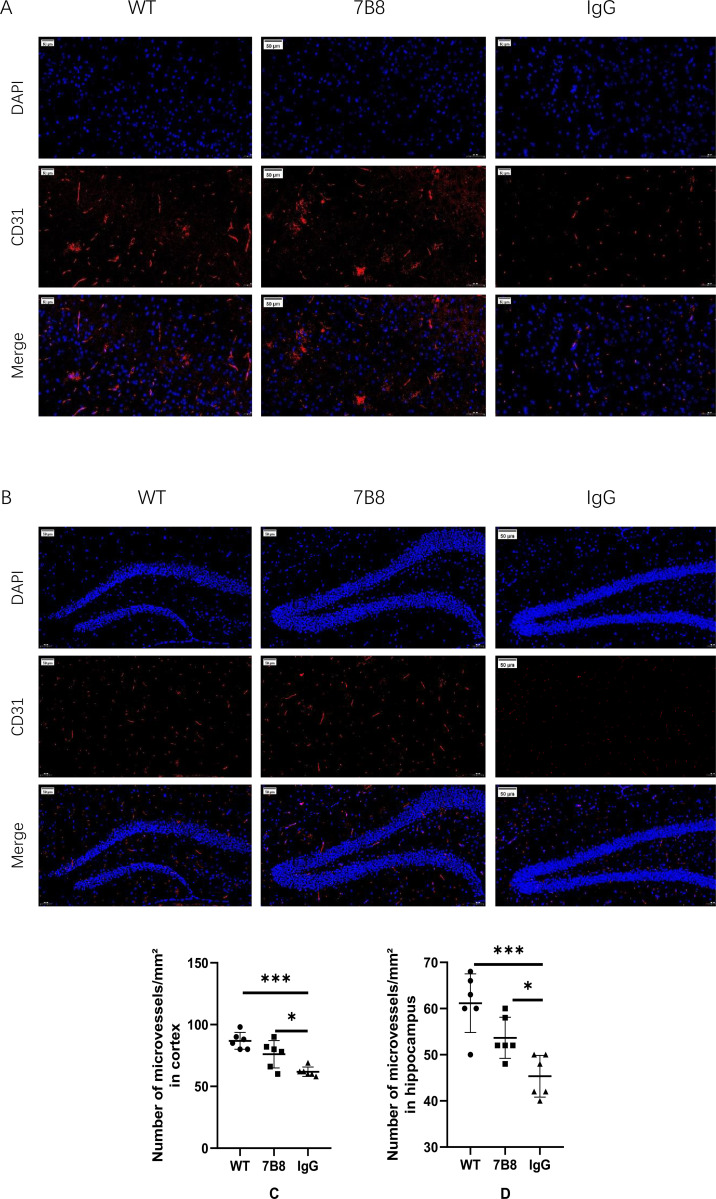
Protective effect of antibody 7B8 on endothelial cells. **(A)** IF staining of endothelial cells in cortex of 7B8 group, IgG group and WT group. (scale: 50μm) **(B)** If staining of endothelial cells in hippocampus of 7B8 group, IgG group and WT group. (scale: 50μm) **(C)** The number of CD31-positive microvascular in the cortex of IgG group was significantly lower than that in WT group and 7B8 group (n=6, *P<0.05, ***P<0.001). **(D)** The number of CD31-positive microvascular in hippocampus of IgG group was significantly lower than that in WT group and 7B8 group (n=6, *P<0.05, ***P<0.001).

CD31-positive microvascular endothelial cells were significantly more abundant in the cortex (**Figures 6A, C**) and hippocampus (**Figures 6B, D**) of both the WT and 7B8 groups compared to the IgG control group (n=6, P<0.05). These findings indicated that the 7B8 antibody exerted distinct protective effects on microvascular endothelial cells—a result consistent with our previous observations of reduced perivascular Aβ deposition and preserved vascular wall structure in antibody-treated mice.

We did not have additional WB data for CD31 protein expression. Future studies will incorporate CD31 WB analysis to further verify vascular integrity at the protein level.

### 2.7 Antibody 7B8 regulated cerebrovascular RAGE and LRP-1 levels

Brain endothelial cells play two pivotal roles in Aβ homeostasis: they form the structural foundation of the BBB via tight junction proteins, and regulate Aβ transport and clearance through membrane-localized receptors—receptor for advanced glycation end products (RAGE) and low-density lipoprotein receptor-related protein 1 (LRP-1). Specifically, RAGE mediates the transport of Aβ from the bloodstream into the brain parenchyma, while LRP-1 facilitates Aβ efflux from the brain to the systemic circulation. Together, these two receptors serve as key regulators for maintaining cerebral Aβ homeostasis.

**Figure 7:**
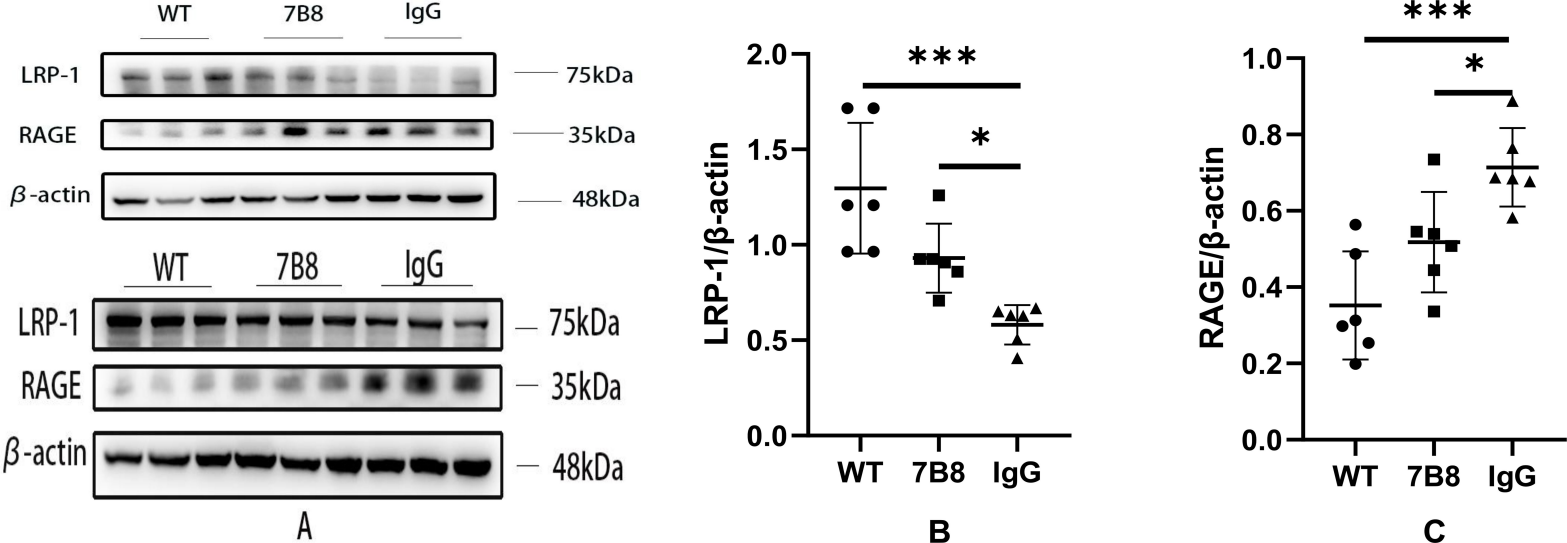
**(A)** The effects of antibody 7B8 on the expression level of RAGE and LRP-1 in vessels of APP/PS1 double-transgenic mice. **(B, C)** WB indicated that LRP-1 level in IgG group was significantly lower than that in 7B8 group and WT group (n=6, *P < 0.05). The RAGE level in IgG group was significantly higher than that in 7B8 group and WT group (n=6, *P<0.05, ***P<0.001).

To investigate whether the 7B8 antibody modulates this receptor balance, vascular fractions were isolated from brain tissue samples, and the expression levels of RAGE and LRP-1 were quantified by WB analysis. The results demonstrated that RAGE expression was significantly higher in the IgG control group than in both the WT and 7B8 groups (n=6,*P<0.05,***P<0.001, **Figures 7A, C**), whereas LRP-1 expression was notably lower in the IgG control group by comparison (n=6,*P<0.05,***P<0.001, **Figures 7A, B**).

Collectively, this receptor expression pattern—decreased RAGE and increased LRP-1 in the 7B8 group—strongly indicated that the 7B8 antibody enhances Aβ clearance by fine-tuning vascular wall function through the modulation of these key transport receptors. This finding is consistent with our previous observation that 7B8 preserves microvascular endothelial cell integrity, suggesting that the antibody’s vascular protective effects are a key contributor to its overall reduction of cerebral Aβ deposition. While direct confirmation of this mechanism would require more targeted assays (e.g., receptor binding assays), the current data align well with this proposed regulatory pathway.

### 2.8 Antibody 7B8 protected tight junction proteins

**Figure 8:**
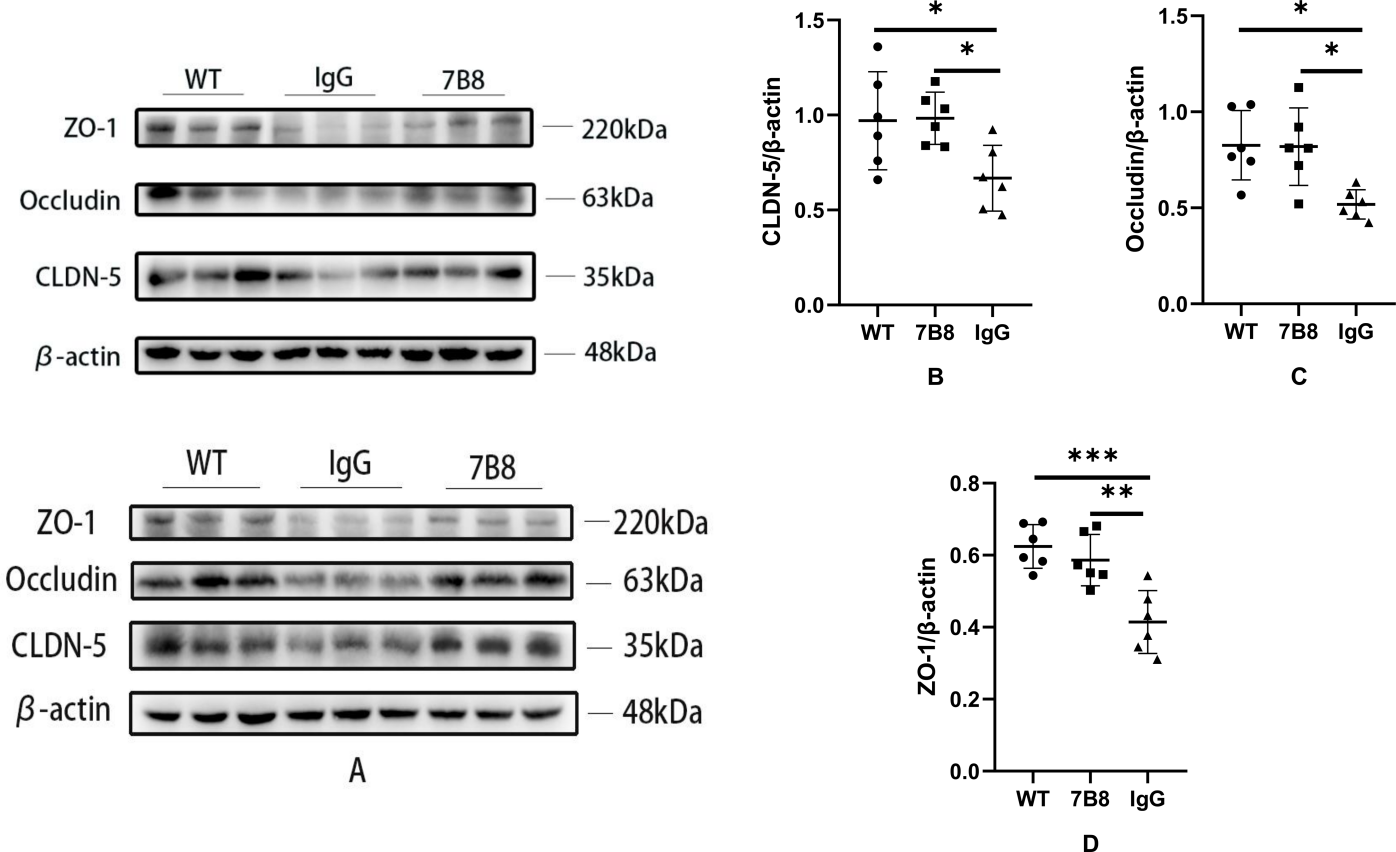
Protective effect of antibody 7B8 on tight junction protein of BBB in APP/PS1 transgenic mice. **(A)** The tight junction proteins (CLDN-5, Occludin and ZO-1) in the 7B8 group, IgG group and WT group were detected by WB. **(B–D)** Comparison of gray intensity of tight junction proteins CLDN-5, Occludin and ZO-1 in 7B8 group, IgG group and WT group, the gray intensity of tight junction proteins in IgG group was significantly lower than that in WT group and 7B8 group (n=6, *P<0.05, **P<0.01, ***P<0.001).

Notably, tight junction proteins serve as the critical structural basis for sealing the paracellular space between brain endothelial cells, playing an indispensable role in preserving the structural integrity and functional stability of the BBB. To investigate whether the 7B8 antibody protects these key proteins in APP/PS1 mice, WB analysis was performed to quantify the expression levels of three major tight junction proteins—zona occludens-1 (ZO-1), Claudin-5 (CLDN-5), and Occludin (**Figures 8A, C**)—in brain tissue homogenates. The results demonstrated that the expression levels of CLDN-5 (**Figures 8A, B**), ZO-1 (**Figures 8A, D**), and Occludin (**Figures 8A, C**) were significantly higher in both the WT and 7B8 groups compared to the IgG control group (n=6,*P<0.05,**P<0.01,***P<0.001, **Figure 8**). These findings indicated that the 7B8 antibody mitigated the impairment of tight junction proteins and thereby contributes to safeguarding BBB integrity. Consistent with our previous observations—including the preservation of microvascular endothelial cells and modulation of Aβ transport receptors—the elevated expression of these tight junction proteins confirms that the 7B8 antibody exerted multi-faceted protective effects on the BBB. Specifically, its benefits extend beyond Aβ clearance to strengthening the structural foundation that maintains the stability of the brain’s microenvironment. While direct confirmation of this protective effect would require additional studies (e.g., immunofluorescence staining to visualize the localization and integrity of tight junction proteins), the current WB data align well with our overall research hypothesis.

### 2.9 Antibody 7B8 passive immunotherapy reduced glial cell activation in APP/PS1 mice

**Figure 9:**
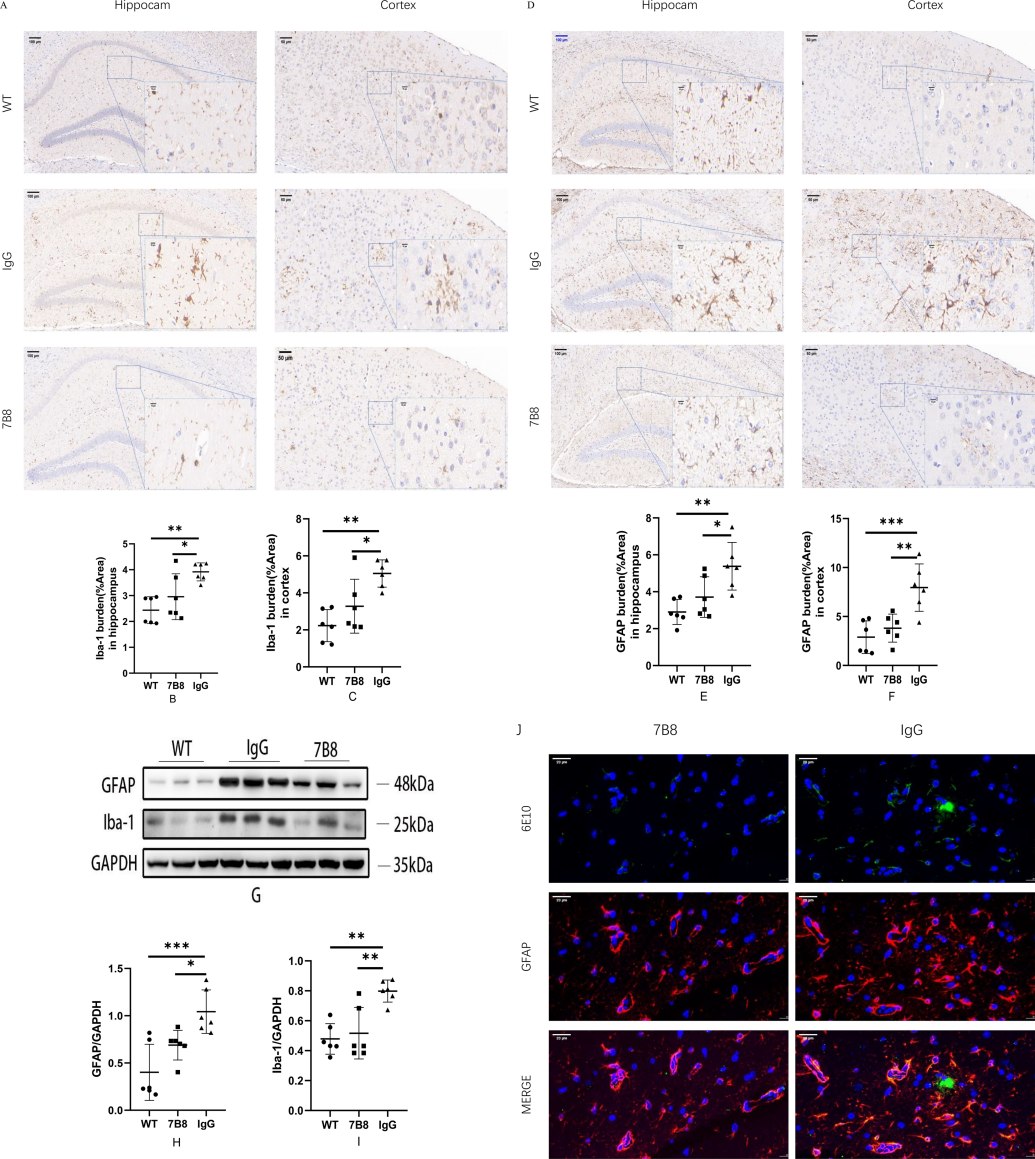
The activation of glial cells in brain tissue was detected by IHC、IF staining and WB. **(A–C)** Compared with WT group and 7B8 group, the number of activated microglia in IgG group was significantly increased (n=6, *P < 0.05, ** P < 0.01). (scale: 50μm in cortex; 100μm in hippocampus) **(D–F)** Compared with WT group and 7B8 group, the number of activated astrocytes in IgG group was significantly increased (n=6, *P<0.05, **P<0.01, ***P<0.001). (scale: 50μm in cortex; 100μm in hippocampus). **(G–I)** WB detection showed that compared with WT group and 7B8 group, the proportion of GFAP/β-actin and Iba-1/β-actin in IgG group was significantly increased (n=6, *P<0.05, **P<0.01, ***P<0.001). **(J)** The activation of Aβ and peripheral astrocytes was detected by IF staining (scale: 20um).

To investigate the effect of the 7B8 antibody on glial activation in APP/PS1 mice, we assessed the microglial marker Iba-1 and astrocytic marker GFAP using IHC, IF, and Western blotting. Activated microglia and astrocytes in the cortex and hippocampus, as well as GFAP/Iba-1 protein levels, were significantly lower in the 7B8 group than in the IgG control group (n=6, *P<0.05, **P<0.01, ***P<0.001, **Figures 9 A–I**). WT mice were included as a physiological control with baseline low glial activation. IF staining examination further showed markedly higher perivascular astrocyte activation around residual plaques in the hippocampus of the IgG group compared with the 7B8 group (**Figure 9J**), which may be linked to Aβ deposition in the vessel wall and brain parenchyma. Given that Aβ is a key trigger of glial overactivation in AD and 7B8 effectively reduces Aβ burden, these results suggested that 7B8 alleviates excessive glial activation and subsequent neuroinflammation, which may contribute to its neuroprotective effects. Further quantification of pro-inflammatory cytokines will help verify the causal relationship between glial inhibition and neuroprotection.

### 2.10 Antibody 7B8 passive immunotherapy did not elevate inflammatory factors in brain tissue of APP/PS1 mice

**Figure 10:**
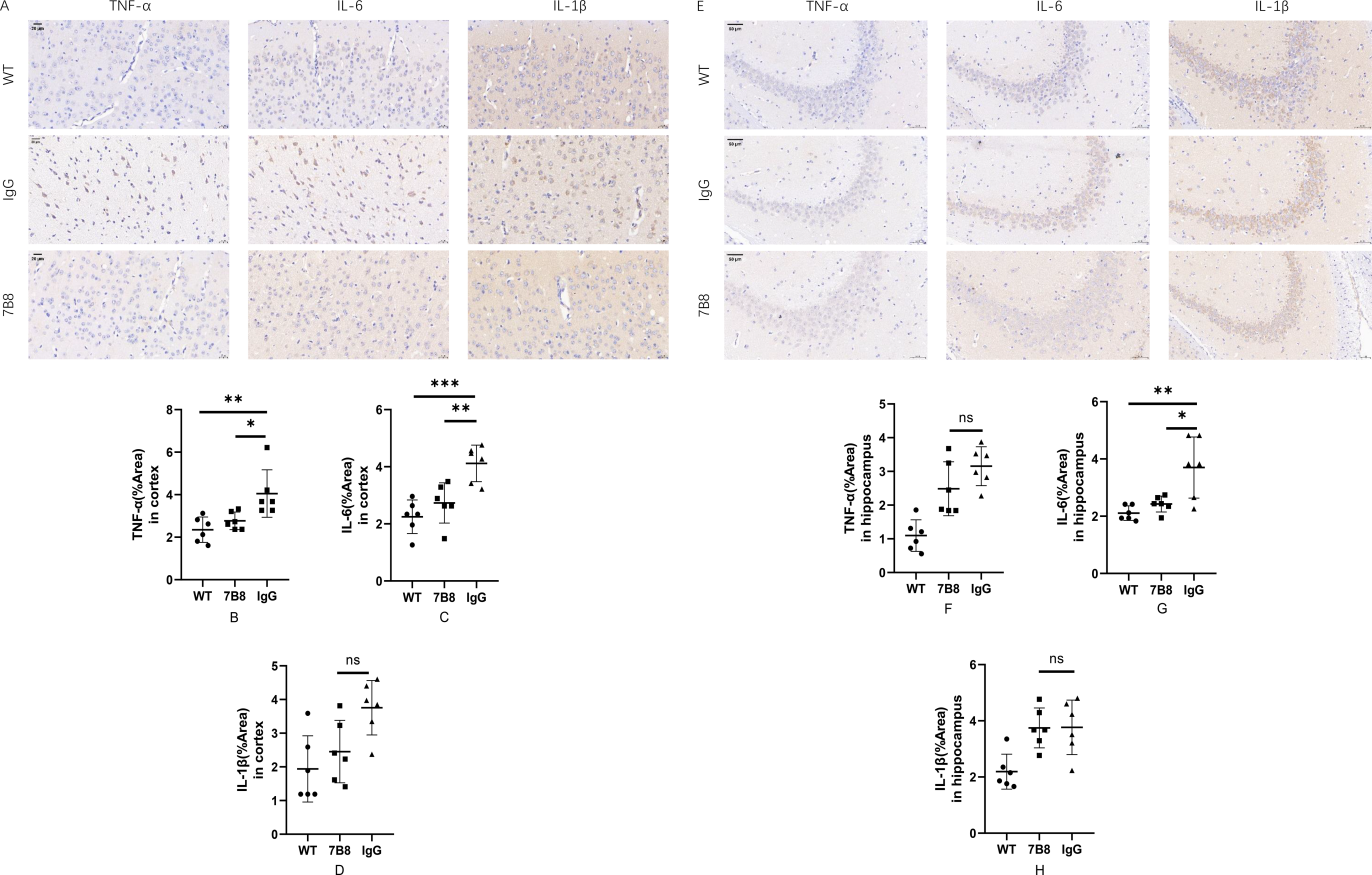
The level of TNF-α, IL-6 and IL-1β were detected by immunohistochemical staining. **(A–D)** The levels of IL-6 and TNF-α in the cortex of IgG group were significantly higher than those of WT group and 7B8 group (n=6, *P<0.05, **P<0.01, ***P<0.001). Scale: 20um. **(E–H)** The level of IL-6 in hippocampus of IgG group was significantly higher than that of WT group and 7B8 group (n=6, *P<0.05, **P<0.01). Scale: 50um.

To clarify the effect of the 7B8 antibody on inflammatory factor levels in APP/PS1 double-transgenic mice, the levels of key proinflammatory cytokines—interleukin-6 (IL-6) and tumor necrosis factor-α (TNF-α)—were quantified across the WT, IgG control, and 7B8 groups. The results revealed a region-specific regulatory pattern: 7B8 immunotherapy significantly reduced IL-6 levels in the hippocampus (n=6, *P<0.05, **P<0.01, **Figures 10E, G**), whereas in the cortex, it decreased both TNF-α and IL-6 levels (n=6, *P<0.05,**P<0.01,***P<0.001, **Figures 10A–C**). No significant differences in cortical and hippocampal IL-1β levels (n=6, P>0.05, **Figures 10D, H**) or hippocampal TNF-α levels (n=6, P>0.05, **Figure 10F**) were found between the 7B8 and IgG groups.

These observations were biologically coherent, as activated microglia and astrocytes are well-documented major producers of IL-6 and TNF-α in AD models—and our prior findings demonstrated that the 7B8 antibody suppresses excessive glial activation. Collectively, these data confirmed that the 7B8 antibody can attenuate the production of proinflammatory cytokines in brain tissue, which is consistent with its inhibitory effect on glial overactivation. Notably, this indicated that the multifaceted benefits of the 7B8 antibody—including Aβ clearance and neuronal protection—may be partly mediated by its suppression of detrimental neuroinflammation. While expanding the panel of detected inflammatory markers (e.g., interleukin-1β [IL-1β]) would provide a more comprehensive understanding of the antibody’s anti-inflammatory profile, these initial results align consistently with our research hypothesis.

### 2.11 Antibody 7B8 inhibited the HMGB-1/RAGE/NF-κB neuroinflammatory signaling pathway in brain tissue

To investigate the effect of the 7B8 antibody on the high mobility group box 1 (HMGB-1)/receptor for advanced glycation end products (RAGE)/nuclear factor-κB (NF-κB) pathway—a canonical neuroinflammatory signaling cascade—we performed WB analysis. Pathway overactivation promotes excessive glial cell activation and proinflammatory cytokine release, ultimately inducing degeneration and dysfunction of neurons and endothelial cells. WB was used to quantify the protein levels of HMGB-1 and RAGE in brain tissue, as well as nuclear and cytoplasmic fractions of NF-κB.

**Figure 11:**
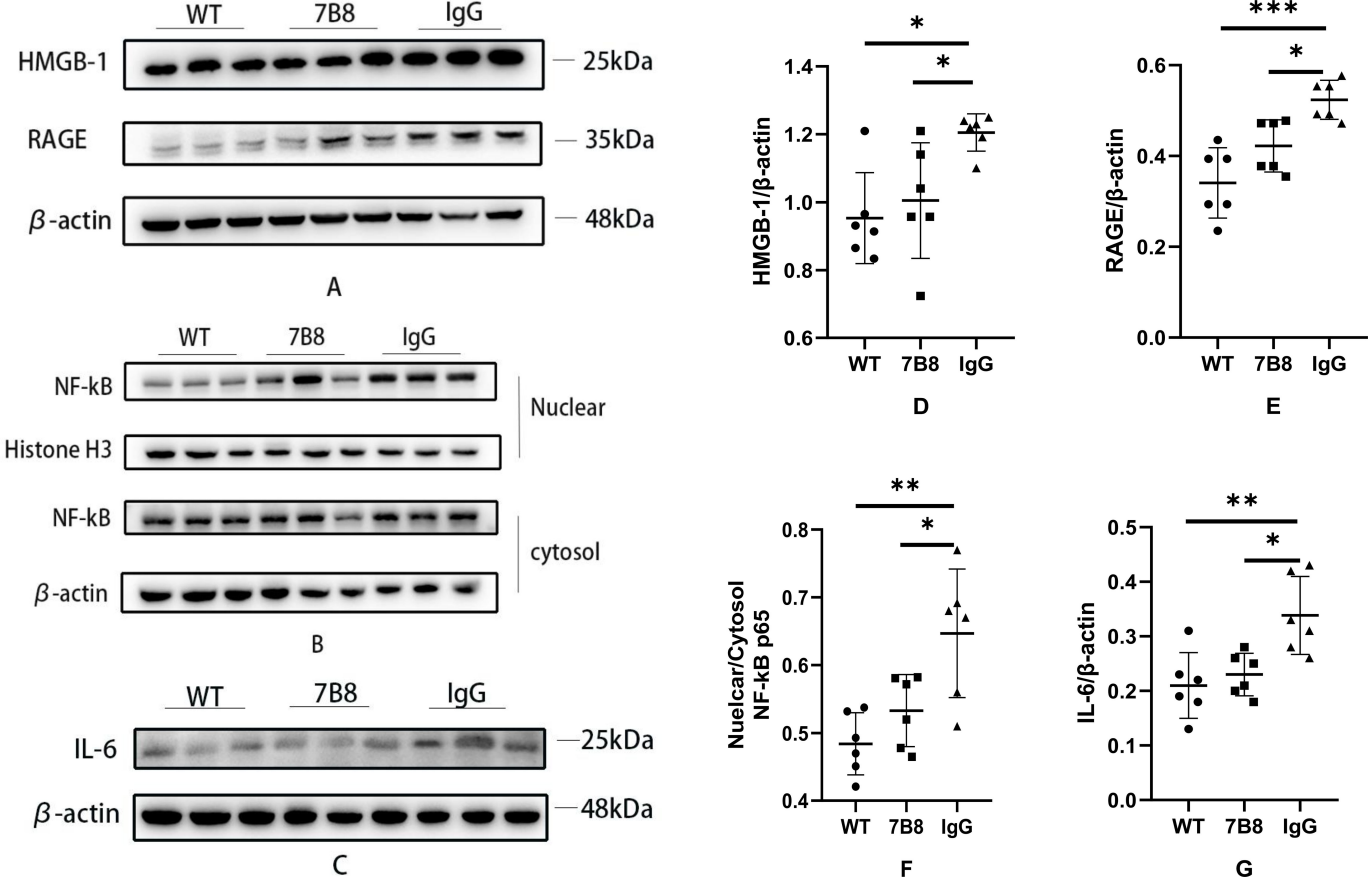
**(A)** The levels of HMGB-1 and RAGE in the brain tissues of WT group, 7B8 group and IgG group were detected by WB. **(B)** The levels of NF-kB in the nucleus and cytoplasm of WT, 7B8 and IgG groups were detected by WB. **(C)** The levels of IL-6 in WT group, 7B8 group and IgG group were detected by WB. **(D–G)** The results of WB indicated that the levels of HMGB-1, RAGE, NF-kB and IL-6 in IgG group were significantly higher than those in 7B8 group and WT group (n=6, *P<0.05, **P<0.01, ***P<0.001).

The results demonstrated that the protein levels of HMGB-1, RAGE, and NF-κB (both nuclear and cytoplasmic fractions) were significantly higher in the IgG control group compared to the WT and 7B8 groups (n=6,*P<0.05, **P<0.01,***P<0.001, **Figures 11A, B, D–F**). Consistent with our earlier findings on inflammatory factors, IL-6 levels were also notably reduced in the 7B8 group relative to the IgG control group (n=6,*P<0.05, **P<0.01, **Figures 11C, G**). Collectively, these data indicated that the 7B8 antibody suppresses glial cell activation and IL-6 release by inhibiting the overactivated HMGB-1/RAGE/NF-κB pathway. This observation was biologically plausible, given the well-documented role of this pathway in driving neuroinflammation in AD models.

Notably, these findings add a critical mechanistic layer to our understanding of the 7B8 antibody’s multi-pronged therapeutic effects: beyond Aβ clearance, the antibody directly targets the downstream neuroinflammatory cascade that exacerbates brain tissue damage. While confirmation of this direct regulatory link would require follow-up studies (e.g., analysis of pathway-related phosphorylation levels or downstream target gene expression), the current data aligns consistently with our overall research hypothesis.

## 3 Discussion

In this study, we used a novel anti-Aβ3–10 monoclonal antibody 7B8 to treat APP/PS1 double-transgenic mice. Previous studies using Aβ1–42 as vaccines for active immunotherapy may cause an autoimmune inflammatory response in the brain, which was caused by T cell epitopes on the C-terminal fragment of the vaccine (28). As the inflammatory response is not only a pathological change during AD, but also a cause of AD (29), vaccines are designed to alleviate autoimmune and inflammatory response side effects by avoiding Aβ15–42 fragments (30). There are two representative antibodies, donanemab (31) and aducanumab (32), whose binding epitopes are both Aβ3–7 monoclonal antibodies. The researches demonstrated that both antibodies can slow cognitive decline in clinical trials without obvious autoimmune side effects. In our previous study, Aβ3–10 fragments were used as vaccines, and this time we developed a novel anti-Aβ3–10 monoclonal antibody from Aβ3-10-KLH actively immunized animals. The Aβ deposition around the vascular wall is mainly fibrillary Aβ, and the previous experiments have confirmed that the anti-Aβ3–10 monoclonal antibody has a strong binding affinity to fibrous Aβ.

Although passive immunization can reduce brain Aβ levels and cognitive impairment in animal models, the efficacy and safety of passive immunotherapy in reducing Aβ deposition around vascular wall are controversial. Previous study found that passive immunotherapy with 3D6 in aged PSAPP mice could reduce Aβ deposition around the vascular wall (33), whereas another study using anti-Aβ3–6 monoclonal antibodies in APP23 mice found no significant changes in the severity or frequency of CAA, but did observe an increased incidence of microhemorrhage (34). Some researchers believed that passive immunity may aggravate the degree of Aβ deposition around the vascular wall and lead to the destruction of vascular structure (35). Aβ deposition around the vessel wall can lead to excessive activation of glial cells, complement activation (13) and a persistent chronic inflammatory response, ultimately leading to the destruction of the permeability of BBB, extravasation of fluid and blood, microhemorrhages and cerebral edema (36). Antibody has a repair effect on the vascular wall structure and will not increase the occurrence of microhemorrhages and cerebral edema (37). In clinical trials of passive immunotherapy, murine monoclonal antibody (mAb) 3D6 was developed and later used in clinical trials in a humanized form called bapineuzumab. Although beneficial effects have been shown in phase II clinical trials, phase III clinical trials of bapineuzumab were terminated early due to insignificant improvements in cognitive function and intracerebral microhemorrhages (38). In clinical trials of aducanumab, although high doses of antibodies can improve cognitive impairment in patients with dementia, ARIA (ARIA-E 30.7%; ARIA-H 30.0%)was also found in MRI detection (39). Meta-analysis suggests that the incidence of ARIA-E and ARIA-H with monoclonal antibodies is 6.5% and 7.8%, respectively (40). Although 80.4% of ARIA are asymptomatic, persistent progression can lead to a variety of symptoms, including headache, nausea, etc. Adverse reactions can be relieved with discontinuation of treatment or dose reduction, but will limit the clinical application of passive immunotherapy at the same time.

Therefore, we urgently need to develop novel antibody to treat AD, which can not only clear Aβ deposits and reduce cognitive impairment effectively, but also avoid microhemorrhage. Based on the results of our previous studies, we developed a novel anti-Aβ3–10 monoclonal antibody 7B8. In order to detect the effect of antibody 7B8 on the Aβ deposits in APP/PS1 mice, we performed immunohistochemical staining. The results showed that the Aβ deposits of hippocampal and cortical in the 7B8 group were significantly less than that in the IgG group, and there were statistical differences (n=6, *P<0.05). WB also showed that antibody 7B8 could significantly reduce Aβ in brain tissue (n=6, *P<0.05). The results also showed that Aβ depositions around vessel wall in 7B8 group were significantly less than that in IgG group (n=6, *P<0.05). Meanwhile, immunofluorescence staining showed that the Aβ depositions around the vessel wall in 7B8 group were significantly less than that in IgG group. The smooth muscle protein of vessel wall in 7B8 group were significantly more than that in IgG group (n=6, *P<0.05).

To evaluate the safety profile of the 7B8 antibody, Prussian blue staining was performed. The results showed no significant difference in microhemorrhage burden between the 7B8-treated and IgG control groups (P>0.05), indicating that 7B8 administration did not induce a marked increase in microhemorrhage formation. Quantification of perivascular Aβ40 deposition confirmed minimal CAA pathology in 6-month-old APP/PS1 mice at both baseline and study endpoint, with comparable CAA vessel counts between the two groups (P>0.05). Notably, the low incidence of microhemorrhages in both treatment groups is most likely driven by the inherently minimal CAA burden at this young age, rather than solely reflecting a direct vascular protective effect of the 7B8 antibody. The favorable safety profile of 7B8 (targeting Aβ3–10) with regard to microhemorrhage risk, in contrast to Aducanumab (targeting Aβ3–7) and Bapineuzumab (targeting Aβ1–5) which are associated with increased microhemorrhages, may be attributed to differences in epitope specificity. Targeting an extended Aβ3–10 epitope may modulate the pattern of vascular Aβ clearance and reduce excessive perivascular inflammatory activation, thereby lowering the risk of antibody-related vascular disruption and microhemorrhage. Future studies using aged APP/PS1 mice (approximately 15 months old) with pronounced CAA pathology will be essential to further verify the long-term safety and efficacy of 7B8, as this model recapitulates the severe vascular amyloid burden more closely resembling human Alzheimer’s disease. Collectively, these findings demonstrate that the 7B8 antibody effectively reduces Aβ deposition in young APP/PS1 mice without exacerbating microhemorrhage formation at this early pathological stage.

In order to detect the effect of antibodies on cognitive function in APP/PS1, we carried out NOR experiments and nestlet shredding test. The results showed that the fossa score was increased after passive immunotherapy, and the NOR experiments showed that the DI was improved after passive immunotherapy (P<0.05 compared with the IgG group), suggesting that monoclonal antibody could decrease cognitive impairment. The antibody 7B8 may alleviate cognitive impairment by alleviating the injury of neurons. The sample size (n=6 per group) was determined based on a prior power analysis (α=0.05, power=80%) and consistent with sample sizes used in previous published studies on anti-Aβ immunotherapy in APP/PS1 mice. We acknowledge that the sample size of 6 animals per group for behavioral assays is relatively modest, a common limitation in preclinical transgenic mouse studies of Alzheimer’s disease due to breeding and experimental resource constraints. While our *a priori* power analysis confirmed sufficient power to detect the key treatment effects of 7B8 observed in this study, future investigations with larger cohorts will further validate the generalizability of these behavioral and pathological findings and help exclude potential weak effects that may be artifacts of limited sample size.

In order to detect the protective effect of antibody on neurons in the brain of APP/PS1 transgenic mice, HE staining and WB detection were performed. The results showed that the damaged neurons in the 7B8 group and WT group were significantly reduced compared with the IgG group, and the expression of NeuN protein was significantly increased (P<0.05), suggesting that monoclonal antibody may improve cognitive function by reducing the damage of neurons.

We now focus on antibody 7B8’s mechanism for clearing Aβ deposits and its impact on vascular wall and BBB structural/functional integrity. LRP-1 and RAGE are the primary receptors mediating Aβ transport across the BBB. In AD patients and animal models, cerebrovascular endothelial cells upregulate RAGE while LRP-1 declines—this impairs cerebral Aβ clearance and drives deposition (41). To assess 7B8’s effect on endothelial Aβ clearance, we extracted mouse cerebral microvessels and measured RAGE and LRP-1 expression. In APP/PS1 double-transgenic mice, 7B8 significantly reduced RAGE levels while increasing LRP-1 (n=6, P<0.05). This suggested 7B8 may inhibit Aβ influx into the brain parenchyma and enhance its efflux to the plasma, boosting endothelial and BBB clearance function. Cerebral capillary endothelial cells form part of the vascular wall and BBB, connected by tight junction proteins to regulate blood-brain substance exchange. To further explore 7B8’s impact on the BBB, we performed immunofluorescence staining for endothelial markers and WB for RAGE, LRP-1, and key tight junction proteins (ZO-1, CLDN-5, Occludin). Immunofluorescence revealed 7B8 reduced endothelial damage in APP/PS1 mice, indicating endothelial protection. WB results showed 7B8 significantly mitigated tight junction protein impairment compared to the IgG group (P<0.05), supporting its role in preserving BBB integrity.

Notably, while the BBB is generally impermeable to large immunoglobulins (molecular weight ~150 kDa), accumulating evidence supports that peripherally administered monoclonal antibodies can access the brain parenchyma via well-characterized pathways, particularly in AD models with pathological BBB dysfunction (42, 43). These pathways include Fcγ receptor-mediated transcytosis across cerebrovascular endothelial cells, low-level constitutive paracellular transport, and enhanced permeability secondary to AD-associated vascular inflammation and tight junction disruption—pathological features that 7B8 itself mitigates as shown herein (44). Although the present study did not directly quantify the exact percentage of 7B8 that crossed the BBB, previous studies on anti-Aβ monoclonal antibodies (e.g., aducanumab, solanezumab) have reported brain parenchymal penetration rates ranging from 0.1% to 1% of peripheral concentrations in AD transgenic mice (45). Critically, even these low levels of brain exposure are sufficient to engage Aβ deposits and modulate vascular endothelial receptors (e.g., LRP-1, RAGE) as observed in our study, given the high affinity of monoclonal antibodies for their targets and the local concentration effects at perivascular and parenchymal Aβ deposition sites (46). Future studies employing isotope labeling or quantitative fluorescence microscopy will enable precise quantification of 7B8’s BBB penetration efficiency and brain distribution.

ARIA can occur in CAA-related inflammation, suggesting it may link to neuroinflammation following passive immunotherapy. Notably, *in vitro* studies show monoclonal antibodies like Gantenerumab and aducanumab activate glial cells (47)—a double-edged effect: while glia clear Aβ via phagocytosis, excessive activation can exacerbate neuroinflammation, damage the BBB, and trigger ARIA (48). *In vivo*, multimodal MRI localized ARIA, and ¹¹C-PK11195 PET (a marker of activated glia) confirmed glial activation around ARIA lesions, reinforcing this association (49). Monoclonal antibodies recruit glia to amyloid plaques via Fc receptors, enhancing phagocytosis and synaptic protection to improve cognition. Yet glial activation itself is an inflammatory response—sustained overactivation may increase BBB permeability and induce ARIA (50).

Additionally, activated glia overexpress proinflammatory cytokines (e.g., IL-1β, IL-6, TNF-α), damaging neurons and vascular walls in AD patients and transgenic mice, and worsening neurodegeneration and vasogenic edema (51). Notably, perivascular astrocytes—an astrocytic subtype lining the cerebral vasculature—are the primary source of complement proteins in the central nervous system, and activated glial cells further upregulate the production of these complement proteins, with complement activation acting synergistically with proinflammatory cytokines to amplify vascular and neuronal injury. Consistent with this, a retrospective clinical study of aducanumab-treated AD patients identified microinfarcts with hemosiderin deposition and robust complement activation in brain regions corresponding to ARIA on MRI (32). Moreover, a recent preclinical study in AD mice demonstrated that passive immunotherapy with the anti-Aβ antibody 3D6 led to increased deposition of cerebral amyloid angiopathy (CAA)-associated complement proteins (C3, C5b-9), which correlated with elevated microhemorrhage burden (52). To explore 7B8’s impact on glial activation, we analyzed GFAP (astrocyte marker) and Iba1 (microglia marker). Immunohistochemistry and Western blotting both showed significantly lower GFAP and Iba1 levels in 7B8-treated APP/PS1 mice than in the IgG group (n=6, *P<0.05). Further immunohistochemical analysis of proinflammatory cytokines revealed 7B8 reduced cortical TNF-α and IL-6, as well as hippocampal IL-6 (n=6, *P<0.05). Collectively, these data suggest 7B8 mitigates glial activation and dampens neuroinflammatory cytokine production in APP/PS1 mice.

HMGB-1/RAGE/NF-κB is a key neuroinflammatory signaling pathway—changes in parenchymal/vascular amyloid plaques and their clearance by antibodies may modulate brain inflammation via this axis. In AD patients and animal models, elevated extracellular HMGB-1 (a marker of neuronal injury/necrosis) acts as an endogenous danger signal. It binds RAGE on glia to regulate their activation and on vascular endothelial cells to modulate Aβ transport (53). Moreover, RAGE activation triggers NF-κB signaling, promoting release of proinflammatory factors (e.g., TNF-α, IL-6, IL-1β, ICAM-1, VCAM) and exacerbating parenchymal/perivascular inflammation (54). To assess 7B8’s impact on this pathway, we performed Western blotting. Compared to the IgG group, 7B8-treated APP/PS1 mice showed significantly reduced HMGB-1 and RAGE expression, as well as decreased nuclear NF-κB levels (n=6, P<0.05). Consistent with these changes, cerebral IL-6 (a core proinflammatory cytokine) was also lower in the 7B8 group. These data demonstrate that 7B8 treatment is associated with suppressed HMGB-1/RAGE/NF-κB signaling, reduced glial activation, and diminished release of proinflammatory mediators (e.g., IL-6, VCAM, ICAM-1), ultimately mitigating cerebral and perivascular inflammation. However, since specific pharmacological inhibitors or genetic tools targeting the HMGB-1/RAGE/NF-κB pathway were not utilized in the current study, we cannot definitively conclude that 7B8 directly regulates this signaling axis. Instead, the observed reductions in HMGB-1, RAGE, and nuclear NF-κB expression may reflect an indirect consequence of diminished amyloid plaque burden: clearance of parenchymal and vascular amyloid by 7B8 could alleviate neuronal injury and secondary inflammatory activation, thereby leading to downstream suppression of this neuroinflammatory pathway. This represents an important limitation of the present study, and future investigations employing pathway-specific inhibitors, activators, or genetic manipulations will be critical to dissect the direct versus indirect regulatory effects of 7B8 on HMGB-1/RAGE/NF-κB signaling.

Taken together, our findings showed antibody 7B8 effectively reduces Aβ deposition in the brain parenchyma and vascular walls, protects neurons, and improves cognitive function in AD animal models—notably without increasing cerebral microhemorrhage. To further clarify its effects on vascular wall structure and BBB integrity, and assess potential risks of microhemorrhage or edema, we extended our investigations. Results indicated that 7B8 preserves vascular and BBB structure while enhancing vascular Aβ clearance. Given that microhemorrhage often links to glia activation-driven inflammation, and long-term antibody exposure might trigger chronic inflammation that impairs cognition and vascular structure, we examined microglial and astrocyte activation. Our data revealed that 7B8 dampens glial activation, reduces proinflammatory cytokine release, and alleviates brain and perivascular inflammation. A potential mechanism underlying these effects may involve the inhibition of HMGB-1/RAGE/NF-κB signaling activity.

Our study has several limitations. First, the present study used young 6-month-old APP/PS1 mice with minimal CAA, which did not fully recapitulate the CAA pathology in advanced AD. Thus, the microhemorrhage safety data cannot be extrapolated to mid-aged/aged AD patients with severe CAA. Second, we did not include 7B8 concentration gradients or a positive antibody control (e.g., Aducanumab)—this means we could not assess how different doses influence efficacy and safety, nor compare 7B8’s strengths and weaknesses to established therapies. Third, we did not characterize microglial subtypes, leaving the protective versus detrimental roles of inflammation unexamined. Additionally, the present study used a relatively small number of animals but included an equal number of male and female mice. Although the small sample size did not provide sufficient statistical power for a formal sex-stratified analysis, visual inspection of all primary and secondary outcomes revealed no obvious trends or substantive differences between male and female animals across the experimental groups. Thus, data from male and female mice were combined for all statistical analyses reported in this study. We aim to address these gaps in future research.

To comprehensively evaluate the safety profile of 7B8, we will conduct a follow-up study using 12–15-month-old mid-aged APP/PS1 mice (with prominent CAA pathology) and additionally adopt APP23 and Tg-SwDI mouse models (Dutch/Iowa mutation, classic CAA models) to systematically assess microhemorrhage, vascular leakage, and ARIA-related endpoints. The follow-up study will include a larger sample size (n≥10 per group) to identify potential sex-specific differences, with microhemorrhage quantified via Prussian blue staining combined with Thioflavin S co-staining for CAA vessels, and CAA severity evaluated by counting perivascular Aβ40-positive vessels per mouse (≥20 high-power fields per sample).

## 4 Method and materials

### 4.1 Animals

Twelve 6-month-old APP/PS1 double-transgenic mice (C57/BL6 background) and six age-matched wild-type (WT) C57/BL6 mice were purchased from Beijing HF Bioscience Co., Ltd. (Beijing, China). Additionally, six 6-month-old untreated APP/PS1 mice (C57/BL6 background, 3 females and 3 males) were included as a baseline control group to evaluate the severity of cerebral amyloid angiopathy (CAA) and amyloid deposition at the onset of immunization. All mice were maintained in the Laboratory Animal Center of China Medical University under specific pathogen-free (SPF) standard conditions: temperature 23 ± 2°C, humidity 50 ± 10%, and a 12-h light/dark cycle, with free access to standard chow and sterile water.

The 12 APP/PS1 mice were randomly assigned to two experimental groups (n=6 per group, 3 females and 3 males) using a random number table: (1) the 7B8 group (treated with anti-A_3–10_ monoclonal antibody) and (2) the IgG group (treated with non-specific isotype control antibody). The WT group (n=6, 3 females and 3 males) served as a negative physiological control, as these mice lack human APP/PS1 transgenes and do not develop Aβ plaques or significant CAA pathology (consistent with previous reports (25, 26)). Notably, 6-month-old APP/PS1 mice are characterized by early-stage parenchymal Aβ deposition but minimal CAA (26), which is suitable for initial evaluation of 7B8’s efficacy in amyloid clearance and preliminary safety (e.g., microhemorrhage risk) in a low-CAA background.

All animal experiments were performed in strict accordance with the Guidelines for the Care and Use of Laboratory Animals of the Animal Care and Use Committee of China Medical University (Approval No.: [CMU2021274]) and complied with the ARRIVE guidelines to ensure animal welfare and experimental rigor.

### 4.2 Anti-Aβ3–10 antibody synthesis

Amino acid sequence: H-Glu-Phe-Arg-His-Asp-Ser-Gly-Tyr-COOH (EFRHDSGY) as Aβ3–10 site, antigenic peptide 5xAβ3–10 and vaccine Aβ3-10-KLH preparation process in cooperation with GeneScript, using Aβ3-10-KLH to immunize 5 mice, collect animal antiserum, preliminary screening using ELISA method, *In vitro* experiments used dot-blot analysis and immunohistochemistry to verify the screening results.

A total of eight immunizations were performed from the age of 6 months at one-week interval. Antibodies (7B8 and IgG) were dissolved in PBS at 2mg/mL and injected into the mice intraperitoneally at 10 mg/kg. WT group mice were kept under the same conditions without additional treatment. Mice were euthanized under anesthesia at 9 months of age.

### 4.3 Nestlet shredding test and novel object recognition experiment

#### 4.3.1 Nestlet shredding test

The general method is as follows: (1) Carry out the experiment on the 3rd day after the last injection, prepare enough 5*5cm size pieces of paper; (2) Put 8 pieces of paper on one side of each mouse cage, and after staying overnight, record the nest of mice and take pictures the next morning, give corresponding scores, and record for a total of 0–7 days; (3) The scoring criteria are: if the pieces of paper are moved and there are slight bite marks, the score is one point; If the pieces of paper are moved and there are obvious signs of being bitten, the score is two; If most pieces of paper are torn into strips, the score is three points; If most pieces of paper are torn and broken and moved to the corner, the score is four; If all pieces of paper are torn and moved to corners, a score of five points. Scores of 4–5 are marked by nestlet shredding behavior.

#### 4.3.2 NOR experiment

The general method is as follows: (1) NOR includes the adaptation period, familiarization period and recognition period, each time the mouse is placed at the beginning of the experiment, the front is facing the fixed corner, and the experimental compartment is wiped with 75% alcohol during each mouse rest; (2) The adaptation period is 2 days, do not put other objects in the test box, put the mouse in the middle of the box, let it adapt to the activity for 10 minutes, then rest for 20 minutes, and repeatedly adapt 3 times; (3) Day 3 is the familiarization period: put 2 identical toys (AA or BB) in the test box. The time the mice spend exploring each object is measured by intermittent sums; (4) Identification test period: put another object into the test box before replacing a toy, and place the mouse at an equal distance from the two objects for 10 min; (5) The time spent exploring new and old objects can evaluate the recognition ability of mice to objects, and the discrimination index (DI) is calculated by exploring time; The DI calculation formula is: DI=(N-F)/(N+F)*100% means that N(new) is the time spent exploring new objects, and F(familiar) is the time spent exploring old objects.

### 4.4 Tissue preparation immunohistochemistry to detect Aβ burden

After the experiment, the mice were anesthetized with an intraperitoneal injection of 30 mg/kg sodium pentobarbital, followed by sacrifice under deep anesthesia. Subsequently, transcardial perfusion with physiological saline was performed, and the brains were immediately harvested for subsequent analyses. The left hemisphere was fixed in 4% paraformaldehyde for 48h and the right hemisphere was stored at -80 °C. The left hemisphere was infiltrated into liquid paraffin at 70°C for 90 min and finally embedded in paraffin blocks. The paraffinized tissue samples were sliced into 4-μm-thick sections. The sample sections were incubated with antibody 4G8 (SIG-39200; Bio Legend, USA) at 4°C. Then, they were incubated with biotinylated goat anti-mouse IgG secondary antibodies and then incubated with anti-mouse IgG conjugated to HRP, followed by 0.025% DAB. We observed the images with confocal laser scanning microscope and analyzed the representative images with Image-Pro plus 6.0.

### 4.5 Immunofluorescence

Paraffin sections were sequentially deparaffinized in three grades of environment-friendly deparaffinizing solution (15 min each), rehydrated through anhydrous ethanol (twice for 5 min each), 85% and 75% ethanol (5 min each), and rinsed with distilled water; they were then subjected to antigen retrieval in EDTA buffer (pH 8.0) via microwave (8 min medium power to boiling, 8-min pause, 7 min low-medium power, avoiding drying) and washed three times with PBS on a shaker. After slight air-drying, a hydrophobic circle was drawn, BSA was added for 30-min blocking, and diluted mouse-derived α-SMA (1:200) and CD31 (1:200) primary antibodies were applied for overnight incubation at 4 °C. The next day, sections were washed three times with PBS, incubated with corresponding HRP-conjugated secondary antibody for 50 min at room temperature, treated with TSA in the dark for 10 min after PBS washing, and rinsed three times with TBST; bound antibodies were stripped via microwave under the same retrieval conditions before adding diluted rabbit-derived anti-Aβ40 antibody (1:200) for overnight incubation at 4 °C. Following three PBS washes, sections were incubated with corresponding HRP-conjugated fluorescent secondary antibody for 50 min at room temperature, washed three times with PBS, counterstained with DAPI in the dark for 10 min, treated with autofluorescence quencher for 5 min (followed by 10-min running water rinse), mounted with anti-fluorescence quenching medium, imaged under a fluorescence microscope (DAPI: 330–380 nm excitation/420 nm emission; FITC: 465–495 nm/515–555 nm; CY3: 510–560 nm/590 nm), and analyzed using Image J software.

### 4.6 Image analysis

All microscopic images were acquired using an Olympus BX53 fluorescence microscope equipped with a digital imaging system, and quantitative analysis was performed using ImageJ software (Version 1.53c, National Institutes of Health, Bethesda, MD, USA). For each mouse, 3–5 consecutive coronal brain sections with a fixed interval of 150 μm were selected to avoid regional deviation. Regions of interest (ROIs) including the hippocampus and cerebral cortex were defined in accordance with the standard mouse brain atlas, and the same ROI size and location were applied across all groups. Analysts were blinded to experimental allocation during both image acquisition and quantification.

### 4.7 Western blotting analysis

Cortex and hippocampus were dissected and lysed with RIPA lysis buffer, followed with complete homogenization and centrifugation at 10,000× g and 4°C. After centrifugation, the supernatants were collected and the concentrations of proteins were quantified using BCA assay. Protein samples were then loaded onto SDS-PAGE gels for electrophoresis and transferred onto PVDF membranes. Subsequently, the membranes were blocked with 5% BSA solution at room temperature for 1h. After three thorough rinses with TBST buffer, the membranes were incubated at 4°C overnight with diluted primary antibodies against 6E10(SIG-39300; BioLegend, USA), Anti-NeuN (ab104224; abcam, USA), GAPDH (ab8245; Abcam, USA). The residual primary antibodies were removed by three thorough rinses with TBST buffer solution, and the HRP-conjugated secondary antibodies were added to the membranes followed by 1 h of incubation at room temperature on a shaking incubator. Subsequently, the intensities of protein bands were visualized using chemiluminescent substrate working reagents (Tanon; China) and the relative expression levels of certain proteins were measured and calculated using ImageJ software.

### 4.8 Hematoxylin-eosin staining

The paraffin-embedded tissue sections of left hemisphere were heated at 70°C for 1 h and incubated with xylene for 5min for 3 times. The sections were rehydrated by a graded series of alcohols (absolute alcohol to 95%,80%,70%) for 5 min each grade. The slices were counterstained in Harris-modified hematoxylin solution for 5 min and rinsed with distilled water for 30s for 5 times. Then the sections were dipped in hydrochloric acid alcohol solution for 30s to decolor, and rinsed with distilled water. The stained sections were observed under the microscope (Nikon, Japan) at 100x magnification. Finally, sections were stained in eosin-Phloxine solution for 2 min then followed by static washing for 3 times, dehydrated with ethyl alcohol (70%,80%,90%) for three seconds and absolute alcohol for 5 min for 2 times, cleared in xylene for 5 min for twice. Pictures were taken by DS-U3 microscope (Nikon, Japan).

### 4.9 Prussian blue staining

Paraffin section deparaffinized and rehydrated: put the section into xylene I. 20 minutes - xylene II 20 minutes - absolute ethanol I 5 minutes - absolute ethanol II 5 minutes - 75% alcohol for 5 minutes, distilled water wash 3 times. Equal proportions of Prussian blue dyeing solution A and Prussian blue dyeing solution B were prepared as Prussian blue dyeing solution, and the sections were dyed in the dyeing solution for 1 hour, and then washed twice with distilled water. Then stain with Prussian blue staining solution C for 3 minutes, and then rinse with running water. We put the sections into absolute ethanol I. 5 min - absolute ethanol II for 5 min - absolute ethanol III for 5 min - xylene I. 5 min - xylene II for 5 min for 5 min transparent, neutral gum seal. Pictures were taken by DS-U3 microscope (Nikon, Japan).

### 4.10 Isolation of cerebral vascular tissues

Frozen brain tissues were retrieved from -80 °C storage, thawed on ice, and weighed; the tissues were then minced into small pieces, transferred to pre-prepared microvessel isolation buffer (0.32 M sucrose, 5 mM HEPES, pH 7.4), and homogenized using a Dounce homogenizer. The crude homogenate was centrifuged at 1000× g for 10 min at 4 °C, the supernatant (enriched in neuronal components) was discarded, and the dense white myelin layer atop the pellet was removed. The pellet was resuspended in 3 ml sucrose buffer, centrifuged again at 1000× g for 10 min to eliminate residual myelin, followed by centrifugation at 40g to separate large blood vessels from capillaries. The remaining pellet was washed four times with 1 mL sucrose buffer and centrifuged at 350g for 10 min to obtain enriched cerebral microvessels.

### 4.11 Statistical analysis

All data are presented as the mean ± standard deviation (SD). Unpaired Student’s t-test was used for comparisons between two groups (7B8 and IgG control). For comparisons among three experimental groups (WT, IgG control, and 7B8), one-way analysis of variance (ANOVA) followed by Tukey’s honestly significant difference (HSD) *post-hoc* test was performed to control for the inflated Type I error rate associated with multiple pairwise comparisons. Repeated-measures ANOVA was applied for the nestlet shredding test (**Figure 4B**) to account for longitudinal within-subject variation. All statistical analyses were conducted using SPSS 16.0 (IBM Corp., Armonk, NY, USA), and a two-tailed P value < 0.05 was considered statistically significant.

## Data Availability

The original contributions presented in the study are included in the article/supplementary material, further inquiries can be directed to the corresponding author/s.
